# Leadership and organizational ethics: the three dimensional African perspectives

**DOI:** 10.1186/1472-6939-14-S1-S2

**Published:** 2013-12-19

**Authors:** Jude Mutuku Mathooko

**Affiliations:** 1 The Management University of Africa, PO Box 29677-00100 Nairobi, Kenya

**Keywords:** Bioethics, ethical approaches, leadership styles, Internet, ethics drivers

## Abstract

This paper addresses the past, present and future aspects of African leadership and organizational ethics that have, are and will be key for any organization to sustain its systems and structures. Organizational ethics revolves around written and/or unwritten guidelines, ethical values, principles, rules and standards, that are drawn from the harmonious coexistence with the biosphere and it is how these elements are applied that dictates the style of leadership and the ethical thinking of the leaders. Africa has a wide range of complexities which are compounded by, *inter alia*, tribal divisiveness, selfish leadership, wealth inequality, and massive unemployment. Africans tend to draw their leadership and ethical practices and reflections from the events in the environment with which they have interacted for many years. However, in order to fully address and understand the African perspective in leadership and organizational ethics, a broad comprehension of the African diverse and complex landscape is needed through unravelling of the three dimensional existence of the people. African ethics, developed over time, unifies organizations and leadership since it is part of life and is practised, sub-consciously or unconsciously, by the people as they transform from one practice to the other, and during intergenerational transitions. Globalization, liberalization, technological changes and advancement, and market changes are rapidly transforming the environment in which organizations operate. In such a situation, an effective and true leader cannot be rigid but should be flexible, with the ability to use different leadership styles whenever the situation calls for it. Only those leaders with a three-dimensional perspective live inspiring lives, live with a cause and adopt organizational ethics and leadership styles that will stand the test of time. Despite Africa being the cradle of humankind, leadership and organizational ethics is still in its infancy and wanting, even with the new generation of young leaders. The future outlook of African organizational ethics and leadership is to be found in the intersection of changes in technology, life style, demographics and geopolitics with new trends emerging in global polity and economy.

## Introduction

Africa has been perceived as a continent of complexities, with a wide diversity in terms of climate, topography, culture, peoples, and languages. This hallmark complexity is also compounded by tribal divisiveness, wars, selfish leadership, wealth inequality, corruption and massive unemployment. Any attempt to discuss leadership and organizational ethics including other aspects of life should take this scenario into consideration. There is a need for a broad understanding of the African diverse landscape if the African perspective in leadership and organizational ethics is to be fully addressed and understood. Apparently, emerging leadership styles and organizational ethics in Africa have been influenced by the immediate environment that defines the lives of Africans, their existence and connectivity with each other. Furthermore, African leadership styles and organizational ethics are also influenced by the available resources and their utilization, in most cases by a few, while cautiously adapting to the forces of externalities whose ethics has failed to solve the African leadership crisis and problems of corruption. In a nutshell, Africans tend to draw, consciously or subconsciously, their leadership and ethical practices and reflections from the environment in which they have interacted with for many years and have constituted a complex whole, with a moral inter-relationship between social relations and natural events.

African perspective of organizational ethics also revolves around ethical systems, written and/or unwritten guidelines, ethical values, principles, rules and standards, drawn from the harmonious coexistence with the biosphere. It also evolves in the promotion, defence and protection of life, including maintaining the integrity of the human species, protecting the dignity of the person and protecting nature and diversity with the intent of guiding social and moral behaviour. These tenets are enshrined in the Universal Declaration on Bioethics and Human Rights [1].

Lamentably, the ideas and beliefs of the African society that touch on ethical conduct have not been given elaborate investigation and clarification and, thus, stand in real need of profound and extensive analysis and interpretation. To achieve this, the three dimensional (3D: past, present and future) complexity of leadership and organizational ethics must be unravelled and viewed holistically through contextualizing the international ethics normative instruments so that we can confront and understand the way forward for African ethical reflections. This will consequently impact on leadership and organizational ethics in Africa.

## Organizations and global changes

The world is changing very rapidly and managing this change is probably the single most difficult task managers of organizations are confronted with. This pace cannot be handled by traditional management concepts, organizational structures and systems because the society is stochastic in nature and is no longer stable. When there is accelerated change, more and more novel and first time problems arise, and traditional forms of organizations prove inadequate to manage in the new conditions [2]. Over time, those organizations that respond effectively with the changing environments persist and prosper whereas those which do not eventually perish. It is imperative to grasp the changing external environment in order to understand the internal changes of organizations. Toffler [3] observes that in the next 30-40 years, we must anticipate not a single wave of change, but a series of terrible heaves and shudders, caused by, *inter alia*, population growth, urbanization, the shifting proportions of young and old, technological advances, and economic shifts.

## Leadership and organizational ethics in unpredictable times

As stated earlier, the operating environment of organizations in Africa is transforming itself very rapidly, resulting to rarity of excellent leadership. With diverse emerging issues of ethical nature such as corruption, ethnicity, and xenophobia, among others, Africa needs checks and balances in governance. The evolution of globalization, liberalization, market changes, and technological changes and advancement have forced organizations to redefine their structures, systems, and processes [2], and have undoubtedly influenced organizational ethics and leadership.

The world has become integrated and interdependent as never before, making globalization one of the most powerful and pervasive influences on work places, communities, and lives of people. Globalization appears to be irreversible and continues to counter the existing local, regional, national, legal and, presumably, cultural boundaries that have been seemingly blocking the material, ideological, and social transformation [4]. No country can afford to ignore an increasingly globalizing world where interdependence is the norm and a new society is being built, a network society or information society with a series of new opportunities and consequent problems of widening inequalities. This directly applies when we consider the availability of medical services and technologies.

Besides the abounding opportunities within different societies as a result of globalization, many other issues exist which raise questions of profound ethical concern and importance to the global community. Furthermore, many countries have liberalized their economies, particularly in the form of deregulation of industries and tariff reductions, to enhance the efficiency of their economies. This liberalization has a strong influence on medical services especially where fake cheap medicines and equipment have become available to patients and practitioners, respectively. Without strict regulations, our ethical stature and being stand to be questioned and scrutinized.

Change and complexity also define the nature of the 20^th ^Century technology. Today, we are confronted with high turnover of technologies related to medicine and other disciplines. We are further witnessing a compression of the time scale by which new technology is introduced, with ever shorter intervals between discovery and application [5] and the pace of innovation is extremely rapid. This pace of technological change continues to challenge all organizational strategies and affects all individual lives. The availability of facilities such as telemedicine are limited in the developing countries due to their low income per capita and levels of technological advancement. Needless to say, technological change is a *sine qua non *for not only the survival of an organization but also for maintaining its ethics, competitive edge and growth [2].

Economic situations usually compel humans to think differently based on the intensity of the crisis and their present feelings. Ethical reflections could be channelled, positively or negatively, towards mitigations of such experiences and encounters. In these hard times, obtaining more returns from ones profession is tempting if not being practised. People tend to suspend all ethical guidelines and their call of duty to make ends meet. With the dwindling resources, medical professionals tend to opt for cheap and/or outdated medicines and revert to other related unorthodox practices. However, it is worth reflecting that success for a practitioner is generally a function of value for others. Indeed, the paradigm of ethical development emphasizes on the maximization of good and the creation of greatest goodness for the greatest number, while at the same time embracing peace, good health, abundance and progress in all their forms. Based on the African community structure, it should also be understood that African ethics is weighed on duty and not on rights, thus placing a great deal of emphasis on human welfare. A morality of duty is one that requires each individual to demonstrate concern for the interests of others, resulting to African morality duties trumping rights, not the other way around. However, this ethical arrangement is being challenged by the enlightenment of peoples' rights and the emergence of human rights groups. Another influence on African leadership and organizational ethics is supererogation, which is an act "beyond the call of duty", an act over and above what a person is required to do as a moral agent. This implies that our African moral sensitivities should be extended to all people, irrespective of their cultures or societies. Furthermore, the interests of all the parties involved in any arrangement ought to be considered in determining how to act ethically and professionally. In this regard, focus on how an organization could act ethically to the internal and external stakeholders should be encouraged. An organization should further take into account all the stakeholders, both individual and collective, with whom it deals [6]. We have to appreciate that health care provider organizations interact with many different groups of stakeholders such as patients and their families, care professionals, other professionals and nonprofessional employees among others. To determine the interests of each of these broad categories, we have to be more specific in their composition and their needs. Nonetheless, it must all the time be remembered that the priority is the patient.

## Information and communication technology advancement and e-medicine

Technologies and innovations are rapidly transforming the world in various ways. One the areas which has been impacted heavily by Information and communication technology (ICT) is medical practice and medical care, raising social concerns on these technologies. Globally, health care is undergoing a transformative phase due to the different uses of e-medicine. Many of the African peoples cannot afford specialized medical services and yet globalization will not stop to wait for Africa to catch up with medical services and technologies. ICT, including Internet, ambient devices, and intelligent computer systems, has provided fast expert online medical information, consultations, drug prescription and disease diagnosis [7]. Patients can now consult their doctors, purchase drugs and retrieve their medical records through Internet. Further, ICT has been useful in genetic sequencing, mapping of genes that are responsible for human dispositions, and also in genetic profiling for any defective genes which may result in severe illness or chronic disease. Although Internet has several benefits as cited by Collste *et al. *(online), it also raises several ethical problems such as the information it provides which may not necessarily be beneficial for moral autonomy. The procedure of genetic profiling has raised many ethical questions for scientists and practitioners, especially questions on informed consent and human choice. Furthermore, the consultation of a doctor online affects the patient-doctor relationship [8] which is embedded by values of commitment, trust, privacy, confidentiality and responsibility [7]. For e-medicine to advance, issues of, *inter alia*, autonomy and human choice, privacy and confidentiality, and informed consent should be addressed. This way, medical ethics in organizations would be better managed.

## Approaches to ethics in organizations

There are several approaches to ethics in organizations which may range from *Laissez-faire *to a highly proactive methodology that spells out specific behavioural expectations in detail. Some of the approaches concentrate more on managing values and integrity. An organization that adopts the *Laissez-faire *approach to ethics allows employees and management, with little or without ethical training, to make ethical decisions for themselves based on their own judgments and standards of morality. In this regard, the employees and management are expected to do the "right thing" which is not specifically defined, placing the entire burden for ethical compliance upon the individual or the management. The problem with this approach is that each person may have a different way of defining what is ethical and what is not. If *Laissez-faire *leadership accompanies this ethical approach, then the lowest productivity among the employees and management is experienced. Exhortations ethical approach, on the other hand, employs general exhortations to do the right thing, or to be honest, or to be fair. There is a need for training and frequent urging of employees to behave ethically; this gives little help in complex decisions and implies that employees are to pay the short-term cost of acting ethically. Managing values and integrity is an approach that defines and builds on the organization's values, its aspirations as well as identifying the minimum ethical standards. Leaders who take this approach educate, model ethical behaviour, and reward those who abide by organizational values and standards. At the extreme is the compliance approach where organizational ethics is defined in an extremely detailed manner. In this approach, minimum standards of behaviour and severe or deterrent penalties for violations are specified and established by the organization, including the exact ethical behaviour expected in relationships. The employee Handbook on Code of Conduct and Ethics usually offers a list defining and articulating such ethical actions as, *inter alia*, keeping client confidentiality, and not spreading rumours or gossiping about other employees. Non-compliance has its specific penalties and can be disastrous to the employees. To emphasize on this, some organizations have gone a step further and introduced Compliance Plus. In this, they have programmes to ensure employees obey laws and keep the organization untainted. This is essentially a risk management strategy and often does reduce equally damaging unethical conduct.

## Employees and ethics of an organization

Defining and managing the values of a collective group of people within an organization composes the practical application of organizational ethics. The employees, who are the assets of an organization, should maintain the decorum and ambience of the workplace and should be uniformly treated ethically and professionally, regardless of their race, religion, culture or lifestyles. This is best demonstrated through acts of fairness, compassion, integrity, honour and responsibility. To achieve this, however, all employees should adhere to the organization's guidelines, principles and standards that dictate how individuals should behave inside and outside the workplace. Employees must be made to understand and internalize the standard operating procedures through training. Ethical standards for an organization attempt to define behaviours that produce beneficial effects in the organization and in the organization's sphere of influence, and to avert detrimental behaviours. We should, therefore, eliminate any grey areas that may lead to ethical lapses and detrimental behaviours should be avoided.

## Ethics violations in organizations

Unethical and illegal behaviours committed by people shape the ambiance and character of the organization and these include, *inter alia*, fraud, greediness, corruption, engaging in covert operations, humiliating tactics, sexism, racism, and the rights of others [9]. Kovanic and Johnson [10] state that individual behavior does not exist within a vacuum. This means that one person doing unethical acts can perpetuate a chain of unethical actions in an organization. The principles of autonomy, beneficence, nonmaleficence and justice are at risk for violation in relation to patients, health care professionals, and the general public [11]. Depending on the context of a given code of ethics, penalties and/or sanctions may result from these actions. To deter these violations, organizations have come up with compliance and ethics programmes which inform actions in the organization. In many professions such as medicine, the ethical behavior of the professionals is regulated by codes of conduct and ethics. Even with these codes in place, ethical violations are not uncommon and have resulted in unprofessional conducts. Some of these violations include improper or fraudulent billing practice which is witnessed frequently in public and private hospitals and clinics. In this instance, patients are usually charged for services they did not receive because in most cases the person who receives the bill is often not the person who received the services and cannot be able to authenticate the charges. Similar to this violation is the upcoding of services where the provider submits a bill using a procedure code that yields a higher payment than the code for the actual service rendered. Equally unethical and illegal is practising in a profession without a licence, and without renewing or updating the licences and certifications. Boundary violations are other ethics violations that are encountered in several professions. For instance, in crossing sexual boundaries, both parties may be willing participants but something about the sexual relationship is inappropriate due to the professional's position. Indeed, sexual misconduct and exploitation are extreme boundary violations and are punishable as criminal offences. One example of sexual misconduct is sexual contact with a patient against his or her will and also in the instances where medical officers end up marrying their patients. In some cases, professionals portray conflict of interest which could involve a professional violating the client's trust or places him/her at risk of harm because of dealings with another individual. Many organizations insist on proper handling of documents and files containing sensitive and confidential information; if carelessly handled or not secured according to standards could jeopardize clients' privacy and safety. Conflicts of interest is also another ethical violation and can occur on various levels from the individual to the organization, and other internal and external interactions. This is a rather complex violation; there is need for compliance officers to develop clear policies regarding conflicts of interest and conduct formal reviews of actions and transactions as well as order audits [11]. The main ethical issue involved in conflicts of interest is a breach of trust to the public. This trust has been eroding and has reached all-time low in the recent years [9]. The rapid transformation in health care organizations has been the contributing factor in the erosion of trust. A violation of trust in organizations may prompt verbalization such as angry and sarcastic remarks by personnel, especially if trust has been previously entrenched throughout the organizational levels [12]. Executives abusing power and making self-serving corporate decisions lead to unethical behaviours in organizations [13]. Most of the violations, if not all, are illegal and risk prosecution.

## Leadership style choices

In any organization, the executive has at his/her disposal different styles of leadership to choose from. These include authoritarian, where the leader spends most of the time giving out instructions; democratic/participative, where the leader believes that employees are capable and/or experienced; *Laissez faire*/free reign, where the leader lets the employees have free reign over the approach, the decision making and basically all aspects in getting the job done; charismatic, where the leader infects others with energy and belief and the sheer presence becomes a strong boost to the morale and drive of the employees; and transformational, which is required when radical change is needed, when the future is less than certain, and when the goal is pioneering or trailblazing new ideas/concepts. However, some executives practise trial and error and do not even know what style(s) they could use. True leaders choose style(s) that develop visions which meet the needs of others, and in the fulfilment of that, their own needs are met. Above all these styles, the question which arises is whether one is a pull or a push leader. Pull leadership makes employees energized towards institutional innovation [14]. It is the trigger that drives the passion of a workforce to excel. This is characterized by the ability to communicate so that employees engage with the message and become its bearers, leading to the formation of performance communities within the organization. On the contrary, push leaders often disengage themselves from staff and engage in traditional, one-way communication strategies. Their employees either do not understand what is required of them, or are not passionate about it. Whenever the leadership style that you choose suits the circumstances, you are being a good leader. Apparently, there are a lot of arguments for and against each of the effective leadership styles and there is no consensus on which of the styles is the best. However, from the foregoing, it is apparent that an effective and true leader cannot be rigid but should be flexible, with the ability to use the different leadership styles whenever the situation calls for it. That is the best leadership style.

## Demonstrating ethical and moral leadership

Effective leadership is the core of every successful organization. Consequently, effective leaders are models of ethical and moral leadership. They project integrity and emotional intelligence by promoting and supporting an environment where staff are always trying to do "what is right." Leaders with integrity are focused and purposeful, and are always attentive to being consistent with what they say and what they do. They also demonstrate courage in difficult and challenging situations, and provide a model of moral leadership for others to emulate.

## The three dimensional African perspectives in leadership and organizational ethics

Every human being has the right of existence and choice. This requires that every human being lives, exists, and acts in three dimension (3D) to actualize that right of existence and choice. Consequently, in any organization, there are three categories of people, one category of people that is so consumed with the past and forgets the present; secondly the category too consumed by the present that it forgets the future; and lastly that category which is engrossed in talking about the future that it has no present relevance. It is presumed that these categories will manifest different ethical behaviours. With the existence and acknowledgment of these categories, it is therefore expected that leaders would harness their synergy for the organization to forge forward with its vision. Our ethical and leadership knowledge of the past should be used to live the present and to shape the future. As we use our history to shape our future, we should endeavour to nurture more visionaries than missionaries. Only those leaders with a three-dimensional perspective live inspiring lives and live with a cause, which are some of the attributes of true leaders. African leaders should adopt organizational ethics and leadership styles that will stand the test of time; making their lives a history in the making and the future their story. Their actions today are being drawn in the memory of the society which will pass them on as warnings or as inspirations to other generations.

As we attempt to understand the three dimensional perspective of the African ethics and leadership, we can draw some semblance of the 3D with an analogy by Toffler [3] that categorizes different societal developments in terms of "waves" which may influence the ethics and leadership of organizations. They are categorized as the agriculture revolution which transformed the human history, industrial revolution which transformed the society, and the information revolution which is transforming organizations, societies, and economies. The vast sequence of these transformations is accompanied by a deep shift in beliefs, almost a religious conversion from a trust in permanent, tangible things to a belief that even the intangible, ephemeral electronic blips can be swapped for goods or services. As Kandula states, every wave has a hidden code, a set of rules and principles that run through all its activities like a repeated design. People belonging to one wave challenge and attack the beliefs and principles of the earlier wave. All these phases and the three dimensional living affect leadership and organizational ethics.

## The first dimension: past African leadership and organizational ethics

For many years, philosophers and spiritualists have guided people on ethical ways to live and work [11]. However, for the past 300 years, societies have been caught in a windstorm of change which appears to be gathering more momentum. Times have been and are still hard and complex; people continue to be consumed by turbulence and crises which in turn influence ethics and leadership in organizations. The challenges posed by political, economic and environmental dimensions persist and intensify, setting the stage in which professionals operate and dictates how they relate with each other, professionally and/or ethically. It should be noted that these past experiences and challenges were enhanced by under-development which has been a hallmark of Africa.

Africa is very diverse and has a complex multicultural society which has dictated, consciously or subconsciously, the leadership style and the type of ethics and applications that have evolved over the years. Indeed, the evolution of leadership and ethics involved intimate and consistent interaction and association with the environment from time immemorial; making it futile to place a timeframe on the onset of this evolution. For instance, the association with riverine ecosystems and extraction of their resources over time led to the creation of civilizations that dictated the model(s) of African leadership and ethics. To illustrate and understand the past better and in the context of ethics, I will use the evolution of bioethics, a concept which has elicited incessant debates. Here I argue that bioethics is an old concept which has evolved its rules and regulations from antiquity to the present.

## Bioethics: an old concept that bloomed too late in Africa?

Africa is believed to be the cradle of humankind and could have provided insights into medieval bioethics practices and thinking. Africans missed and squandered this strategic position and an enviable early chance of developing a unique African bioethics based on the then values and beliefs. Instead, Africans got entangled into relentless debates on whether bioethics is a new concept or not. From the onset, my opinion is that bioethics is an old concept that bloomed too late in Africa. This is because the evolution of bioethics is intertwined in the evolution of human communities, employing bioethical approaches in tackling their life challenges. This implies that bioethics is part of life and is practised, consciously, sub-consciously and/or unconsciously, by humans as they transform from one practice to the other, and during intergenerational transitions [15]. When bioethics is viewed in the context of a study, it is a "new" concept in Africa. However, African bioethics, when defined in terms of practices and reflections, is "old" and has been part of the ancestral practices. It should therefore be noted that bioethics is more about actions in relation to human life *vis-á-vis *its relation with the environment. The African cultures are tightly held by communities and each community has its own way of operation; they also think and act collectively as a community and in most cases the autonomy of an individual is eroded by decisions made by a well-structured clique of elders whose decisions are revered and final. Contrasting the decisions of elders was going against the whole community and could lead to severe punishment. Therefore, in reflecting on bioethics in the past traditional African settings, we should look at the form of community leadership and structure. During that era, cultures shaped morality and consequently values that guided personal and communal life.

## The second dimension: present African leadership and organizational ethics

The second dimension, the *present*, examines the current status and challenges of organizational ethics and leadership in Africa. Leaders are being faced with crisis today and a strong character is needed to survive. Presently, there are new emerging approaches to organizational leadership and ethics which also pose a challenge to leaders. Truthful communication is hard to come by these days and this has led to misunderstandings in organizations. Furthermore, good ethical leaders are also hard to find today, but when an organization finds that leader, it invests in that leader for the sake of the organization. Ethical leadership has become an essential part of organizational leadership, largely because of the leadership failure that has occurred in business throughout the world, which has led to character-driven leadership styles [11].

Today, practices and beliefs in Africa have been influenced by a great array of externalities. Human beings often have a tendency to depart from the path that they have known for a long time and embrace new approaches. Through infusion and influence from practices and beliefs from the West, the African leadership and organizational ethics are transformed to conform with the current events. With fast growing development in science and technology and emerging chronic and terminal diseases, in-depth understanding of bioethics is required and should be included in medical training and practices. Africa has comparative advantages in indigenous medical knowledge, biodiversity, and diagnostics development. The prevailing high diversity of communities in Africa also makes universality in the application and legislation of traditional medicine an enormous challenge. Traditional medicine is very often misused due to lack of leadership as well as lack of appropriate evidence-based tools, standards, policies, safeguards, codes of ethics, and codes of practice for assessing and enhancing it. Disease ethics is treated as per traditional beliefs in some communities and some are viewed as curse and no need to seek further medication if the traditional medication fails; other beliefs and religions even bar a sick person from being taken to hospital thus violating human rights pertaining to treatment. Another challenge in traditional medicine is dealing with quacks, whose sole interest is not the patient but money, going against the grain of the stakeholders theory where the patient is the main focus. This calls for good ethical leadership and the harnessing and promotion of the good ethical practices and reflections without infringing on the rights of the individuals and communities, even when confronted by emerging technologies and globalization.

## Current leadership in organizations

Presently, a new generation of young, enlightened leaders has emerged across the African continent. This is the Y-generation. Leadership and the position of the leader has greatly evolved to become a highly specialized function of an organization. Today, much skill, training and experience are required to reflect any self-respecting leaders, making an organization's leader to be influential rather than powerful. Power is no longer the characteristic that leaders want to have; they want to convert that power into influence. The leader is supposed to operate on the employees' desires rather than fear and create influence through inspiration and power games. Further, the current crop of leaders is generally genderless. They have both masculine and feminine qualities wrapped into one and ones gender does not count to make it to the top. The present leaders are also more informal and accessible and answer their own emails and do their blog. They are easier to reach, have Twitter and Facebook accounts or whatever other social media network, and are human. They understand the organization in totality and embrace modern approaches in management.

## Current status of bioethics in Africa

African bioethics, in its present form, has been altered and is now a hybrid bioethics having been adulterated by foreign concepts which are not relevant to ethical practices in Africa. Due to the challenges of, *inter alia*, underdevelopment, poverty, communicable diseases, poor healthcare infrastructure, and differences in belief systems, different bioethical responses, practices and reflections have emerged. Furthermore, Africa is a continent which is very heterogeneous, with numerous ethnic groups each with a unique identity, thus making the resultant bioethics even more complex. Coupled with this is the continuous influence of bioethics by globalization and technological transfer and advancement. In this regard, Africa needs bioethics information, research and education in order to comprehend the magnitude of such external influences.

Lamentably, bioethics research in Africa is in its infancy and very few universities and research institutes are engaged in such research. Apparently, the African leadership has ignored bioethics research, most likely contributing to failure of reaching consensus especially in the use of genetically modified organisms (GMOs) as foods. To advance their research and to be in line with the requirements of various ethical guidelines, some African countries have established research ethics and biosafety committees. Poor management and leadership in research, weak legal frameworks and unclear research environment have contributed to increase in cases of infringement on intellectual property rights. Consequently, issues of inadequate information, sharing of benefits and subsequently informed consent emerge [16,17] and are not uncommon. For instance, some vaccination exercises have been rejected and boycotted in some countries because of inadequate information [18]. Therefore, clear ethical guidelines must be available for any researcher or any persons involved in clinical trials; this requires honest and ethical leadership in research. The relevance of bioethics education in Africa is not in doubt especially with the fast advancement of science, technology and innovations which has led to questionable products. Disorganized curriculum schedules in African universities [19], very few books for teaching and limited number of faculty have hampered progress in bioethics education.

## Recognizing United Nations Educational, Scientific and Cultural Organization's efforts and leadership in ethics

United Nations Educational, Scientific and Cultural Organization (UNESCO), which is a key player in ethics education, has engaged itself in the development of bioethics in Africa, mostly through its Division of Ethics of Science and Technology (Sector for Social and Human Sciences). It has produced several documents useful as reference materials for preparing curricula and teaching of bioethics education and also guidelines and principles on the conduct and application of ethics. Some of the notable materials include, *inter alia*, the Core Curriculum in Bioethics, and the following normative instruments: the Universal Declaration on the Human Genome and Human Rights: From Theory to Practice [20], the International Declaration on Human Genetic Data [21], and the Universal Declaration on Bioethics and Human Rights [1]. Another important reference is the Global Ethics Observatory (GEObs) website. In its endeavour to advance bioethics in the universities world-wide, UNESCO also initiated the Universities' Twinning (UNITWIN) Programme in 1992 in which UNESCO Bioethics Chairs are established. The programme focuses on capacity building through exchange of knowledge and sharing and also enhances UNESCO's visibility and popularity through pedagogy, workshops and conferences.

UNESCO has also facilitated the setting up of institutes and centres to offer leadership in ethics in Africa. One of these centres is the UNESCO Regional Bioethics Documentation and Research Centre in Kenya, inaugurated in 2007 as one of the milestones in UNESCO's efforts to promote bioethics. The centre promotes integrated research in bioethics and creates a platform for collaboration, among other mandates. UNESCO also conducts the Ethics Teacher Training Course which provides training to bioethics teachers with the purpose of enhancing their skills and abilities. In this programme, it aims particularly at training a younger generation of teachers so that ethics teaching programmes in the near future can expand and improve in all member states of UNESCO. Despite this, there is still inadequate number of experts on bioethics and hence the need for enhanced bioethics training in the continent [22]. The extent and current status of bioethics in Africa is unknown and also difficult to ascertain [23]. In fact, there is lack of leadership in the development of bioethics education in Africa as exemplified by the present situation where ethics and bioethics are taught as reduced content in the curricula of medicine and life sciences [19,24].

## The third dimension: the future outlook

The African society, facing momentous challenges at the beginning of the new millennium, needs visions of the future so attractive, inspiring and compelling that people will shift from their current mindset of focussing on managing crises to anticipating the future [2]. With the external challenges and the changes that are taking place in organizations' environment, it is imperative that survival strategies should be invoked by leaders so that the future could be taken care of. With this in mind, it is envisaged that the future outlook of organizational ethics and leadership is found in the intersection of changes in technology, life style, regulation, demographics and geopolitics [25] with new trends emerging in global polity and economy, the critical players of change [26]. Furthermore, history has proved, time and again, that the process of change is inevitable in the progress of mankind [27]. With the ever emerging technologies and globalization, accompanied by rapid obsolescence, foreseeing a more improved organizational ethics and leadership in Africa is daunting, even with the new generation and pool of young leaders. However, in whatever situation we find ourselves in, a forewarning was sounded in 2007 by the then Director-General of UNESCO: "Africa- ignore bioethics at your peril." Indeed, bioethics is life and about life, it is not part of life.

## Strategies for promoting future organizational ethics and leadership in Africa

In order to advance future organizational ethics and leadership in Africa, we should engage strategies that can promote uptake and transformative ethical reflections in our complex socioeconomic landscape. This requires that any form of future information and strategy communicating ethical issues must be selected and dispatched in a form that facilitates uptake and understanding at all levels of our society. The goal of future ethics education should therefore be to provide a guide that will give the correct judgment and direction for challenges and dilemmas which African people are confronted with in their daily lives. This will require a cascaded education system where everyone, irrespective of professional background, can intelligently confront and deal with any ethical encounters. In line with this, future ethics content for different education levels should reflect the thinking, traditions and culture of the African people. With bioethics application becoming more apparent and its relevance transcending all spheres of life and the existence of the human race and biosphere generally, it is time bioethics education is made a compulsory course in universities and other institutions in Africa. Its inclusion in the curriculum will also help in confronting bioethical dilemmas in modern and traditional medicine, female genital mutilation, abortion, and environmental degradation which Africa faces today. In order to enhance the use of traditional medicine, the practitioners in this field must have some background in modern medicine; requiring education on the application and use of this type of medicine. It is further emphasized that standard operating procedures should be developed to allow for a wider global view and acceptability of traditional medicine.

## The unfinished business: strategies for promoting bioethics in Africa

For any meaningful future development and application of bioethics to be realized in Africa, organizations should put in place strategies, especially in medical schools and other organizations, that could promote bioethics, some of these strategies include human resource capacity development, training on normative instruments, research and adoption of modern technologies, expansion and diversification of programmes in universities to include bioethics, and the sharing of resources. Inclusion of ethics and/or bioethics education or its branches in the university programmes is necessary to build a critical mass for ethics trainees and faculty. African governments should further embark on training of teachers with the ability to develop quality ethics curricular, to analyze ethical dilemmas which require rational approaches, and to provide authoritative leadership in this area. In addition, training on the various ethical standards, contents of normative instruments, oaths, and organizational and professional Codes of Conduct and Ethics provided in medicine and other related disciplines is required for proper leadership in these disciplines. Medical practitioners need to embrace modern technologies available in the market which include wireless health (telemedicine, portable medical scanners). However, the future in Africa is cloudy due to the low research levels, low innovations in technology and low broadbands for effective utilization of these technological advances.

African countries lag behind in bioethical resources and in order to inculcate ethics in organizations, sharing of ethics materials and new pedagogical approaches amongst institutions should be encouraged. For instance, the UNESCO Bioethics chairs under the UNESCO UNITWIN Programme should be encouraged to have exchange visits to learn from each other and to exchange ideas and share experiences in teaching and examining bioethics.

With the above strategies, African institutions and organizations will still face challenges especially when analyzing a wide diversity of dilemmas due to the diverse African population with different cultural backgrounds and languages. More daunting, however, is cascading ethics to lower levels of the education systems since it requires ingenuous approaches, strategies and focused leadership. In order to offer the required leadership in organizations, we should be abreast with the emerging issues in the field of ethics. Therefore, employees should be facilitated to attend and participate in ethics conferences and workshops since this enhances ethical thinking and practice. Institutions should also be supported to organize meetings, conferences and workshops in order to enhance and strengthen ethical reflections within the institutions and globally.

## Conclusions

Despite Africa being the cradle of humankind, leadership and organizational ethics is still in its infancy and wanting. In cases where standard operating procedures are available, they are not enforced fully and ethical violations still persist and continue unabated. This makes the landscape in which professionals operate very challenging. Even if Africa moves at the pace of the front runners in technologies, this would require full marshalling of the continent's limited resources. Capacity building for critical mass in leadership and application of ethics in organizations is likely to take longer than expected due to the ever changing economic, scientific and technological environment. Universities should take the lead in training and elaborating ethical issues and assisting governments and practitioners in the interpretation of ethical dilemmas which are encountered during the course of duty. However, the diversity of African geographical landscapes and the multiplicity of education systems in Africa coupled with a wide ethnic diversity makes the interpretation of ethical dilemmas extremely complex. With the proliferation of medical schools, leadership and organizational ethics become the unifying forces in the provision of seamless health services. To ameliorate the challenges of leadership and ethics, medical practitioners should be exposed to a compulsory well-regulated and managed ethics education programme.

## List of abbreviations

3D: three dimensions (past, present and future); GMO: genetically modified organisms; UNESCO: United Nations Educational, Scientific and Cultural Organization; GEObs: Global Ethics Observatory; UNITWIN: Universities' Twinning.

## Competing interests

The author declares that he has no competing interests.

## Declarations

Publication of this supplement has been funded by the College of Health Sciences and the Research Office at the University of Kwazulu-Natal.

This article has been published as part of *BMC Medical Ethics *Volume 14 Supplement 1, 2013: Selected papers from the 3rd Ethics, Human Rights and Medical Law Conference (3rd EHMRL). The full contents of the supplement are available online at http://www.biomedcentral.com/bmcmedethics/supplements/14/S1.
